# Differential Contribution of Acute and Chronic Inflammation to the Development of Murine Mammary 4T1 Tumors

**DOI:** 10.1371/journal.pone.0130809

**Published:** 2015-07-09

**Authors:** Celso Tarso Rodrigues Viana, Pollyana Ribeiro Castro, Suzane Motta Marques, Miriam Teresa Paz Lopes, Ricardo Gonçalves, Paula Peixoto Campos, Silvia Passos Andrade

**Affiliations:** 1 Department of Physiology and Biophysics, Institute of Biological Sciences, Federal University of Minas Gerais, Belo Horizonte, Minas Gerais, Brazil; 2 Department of Pharmacology, Institute of Biological Sciences, Federal University of Minas Gerais, Belo Horizonte, Minas Gerais, Brazil; 3 Department of General Pathology, Institute of Biological Sciences, Federal University of Minas Gerais, Belo Horizonte, Minas Gerais, Brazil; University of Sydney, AUSTRALIA

## Abstract

Based on the notion that inflammation favors tumorigenesis, our experiments comparatively assessed the influence of acute and chronic inflammation on the development of a murine mammary tumor (4T1). In addition, we characterized angiogenic and inflammatory markers in the tumor tissue and systemically. Subcutaneous implantation of polyether-polyurethane sponge discs in Balb/c mice was used to host 4T1 tumor cells (1x10^6^), which were inoculated intraimplant 24h or 10 days post implantation. Flow cytometric analysis of enzyme-digested implants revealed that, after 24 hours, the population of leukocytes was primarily characterized by neutrophils (42.53% +/- 8.45) and monocytes (37.53% +/- 7.48), with some lymphocytes (16.27% +/- 4.0) and a few dendritic cells (1.82% +/- 0.36). At 10 days, macrophages were predominant (37.10% +/- 4.54), followed by lymphocytes (28.1% +/- 4.77), and monocytes (22.33% +/- 3.05), with some dendritic cells (13.60% +/- 0.55) and neutrophils (11.07% +/- 2.27). A mammary tumor grown in a chronic inflammatory environment was 2-fold when compared with one grown in acute inflammation and 5-fold when compared with tumor alone. The levels of pro-angiogenic cytokine (VEGF-Vascular Endothelial Growth Factor) were higher in implant-bearing tumor when 4T1 cells were grown in 10-day old implants as compared to the VEGF levels of the two other groups. Overall, the levels of the inflammatory markers evaluated (NAG -N-acetylglucosaminidase, TNF-α –Tumor Necrosis Factor- α) were higher in both groups of implant-bearing tumors and in serum from those animals when compared with the tumor alone levels. This inflammation-related difference in tumor growth may provide new insights into the contribution of different inflammatory cell populations to cancer progression.

## Introduction

Compelling evidence has indicated that inflammation in neoplastic progression plays a decisive role. This concept has been built on a prominent association between persistent inflammatory processes due to parasites, viruses, bacterial infections, and carcinogenesis that occur in a number of organs and tissues [1–5].

Further support for this notion comes from the fact that the inflammatory tumor microenvironment is characterized by the presence of inflammatory cells (macrophages, neutrophils, lymphocytes, eosinophils, and mast cells). These cell populations, together with tumor and stromal cells, secrete a wide range of cytokines, chemokines, and growth factors that directly or indirectly contribute to tumor development [1, 2, 4]. Among the cytokines, VEGF, for instance, is required for the pathological growth of vessels in many conditions including inflammation, retinopathies, and tumors [6]. TNF-α, a major mediator of inflammation, is a tumor promoter factor contributing to stromal development, inflammation, and tumor spread, particularly when chronically produced [7, 8]. Another cytokine, chemokine, CCL2, is responsible for recruiting inflammatory monocytes to the tumor site. The expression of this chemokine and macrophage infiltration is correlated with poor prognosis and metastasis in human breast cancer [9, 10]

The association between inflammation and tumorigenesis has also been demonstrated using experimental strategies in which distinct tumor cell lines and different populations of inflammatory cells are co-cultivated in *in vitro* and/or *in vivo* systems. For instance, implantation of foreign body material has been shown to induce a local inflammatory response in which sarcoma development occurred [11–14].

In 1992, we established an in vivo model in which the sequential development of tumors derived from murine colon 26 adenocarcinoma and B16 melanoma tumor cells could be monitored when the tumor cells were hosted in a synthetic sponge matrix (polyether polyurethane) in mice [15]. Interestingly, the expression of iNOS (inducible nitric oxide synthase), an inflammatory marker, was shown to be more pronounced in tumor-bearing implants when compared with that in sponge implants or tumor alone, implying that the inflammatory process induced by the foreign body reaction intensified inflammation in tumors [16]. This experimental system was further exploited to study the contribution of sponge-induced inflammation to mammary tumor growth. In that study, the growth of tumor cells hosted in subcutaneous implants was delayed when the animals were treated with dexamethasone [17]. In another series of publications, the contribution of inflammation to tumor development was shown using a similar approach. In one of those studies, a clone (QR32) derived from fibrosarcoma cells became tumorigenic and metastatic when subcutaneously co-implanted with a gelatin sponge in mice. Furthermore, inflammation-promoted tumor progression was inhibited by administering an anti-granulocyte antibody [13, 18]. While these studies have contributed to confirming a positive association between inflammation and tumorigenesis, there is a lack of information regarding the influence of acute versus chronic inflamed environments on key components of tumor tissue (angiogenesis, inflammatory cell recruitment/activation, and on cytokine production) and on systemic levels of pro-inflammatory markers. Accordingly, we applied a model of sponge-induced inflammation to host the murine mammary tumor cell (4T1) specifically to examine the influence of the distinct inflammation phases on various murine tumor components and the impact of the malignancy systemically.

## Materials and Methods

### Ethics Statement

The use of animals and procedures for this study was approved by the Ethics Committee of Animal Experimentation (CEUA) of Federal University of Minas Gerais, (protocol number 45/14). All surgery was performed under ketamine and xylazine anesthesia (60 mg/kg and 10 mg/kg, respectively) and every effort was made to minimize suffering.

### Animals

We used male Balb/c laboratory mice weighing 20 g provided by Centro de Bioterismo (CEBIO) of Universidade Federal de Minas Gerais (UFMG). The animals were housed in polypropylene cages inside a well-ventilated room, provided with chow pellets and water *ad libitum* and maintained under a 12-hour light/dark cycle.

### Preparation of sponge discs and implantation

**Fig 1:**
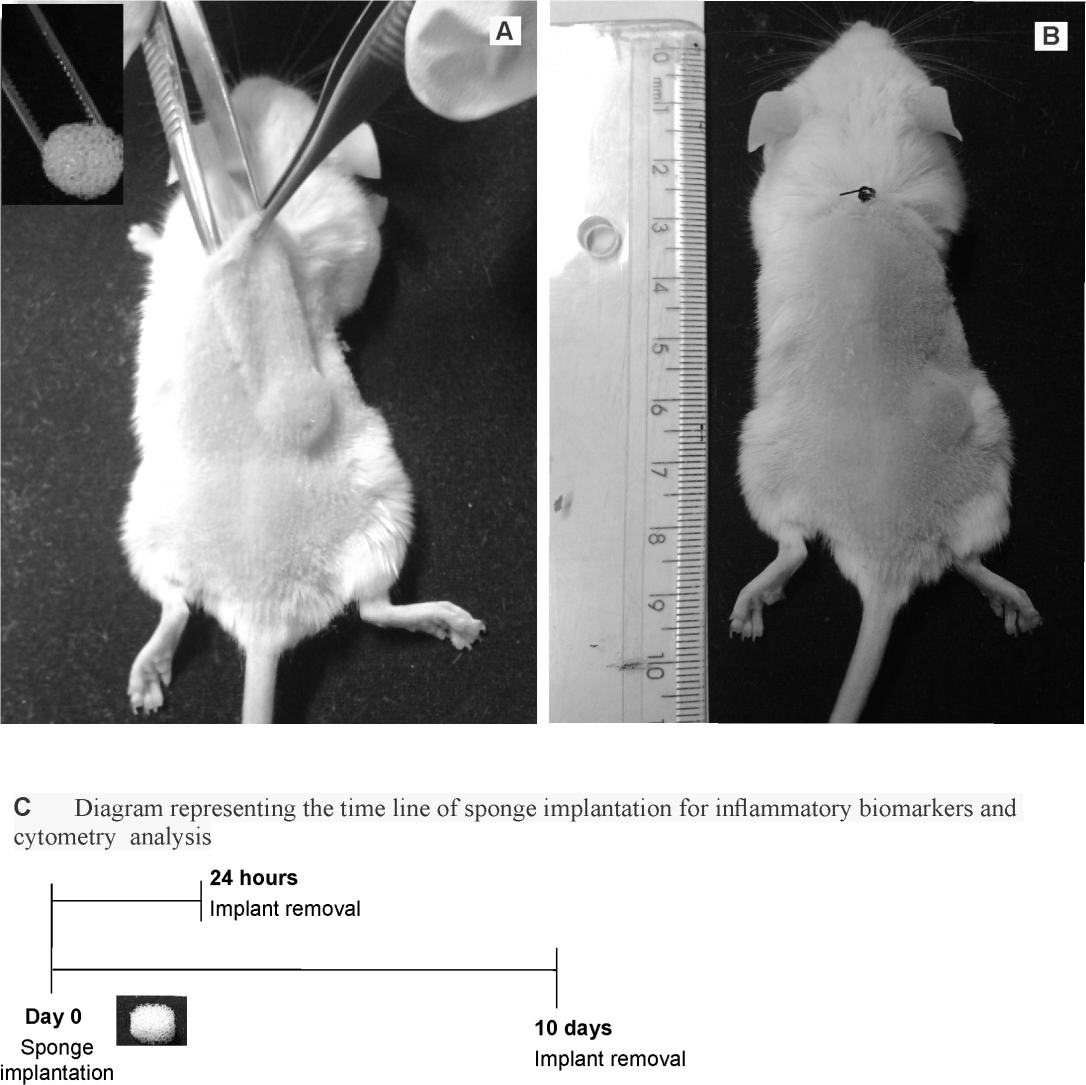
Sponge implant model and diagrammatic timeline of the experiments. Implantation of polyether polyurethane sponges in the subcutaneous space in the back of Balb/c mice (A and B). Diagram representing the time line of sponge implantation for inflammatory biomarkers and cytometry analysis (C).

Polyether-polyurethane sponge (Vitafoam Ltd. Manchester, UK) was used as the implanted material to provide the inflammatory environment to host the tumor cells. The sponge discs, 5mm thick×8mm diameter, were soaked overnight in 70% v/v ethanol and sterilized by boiling in distilled water for 15min before implantation. The animals were then anesthetized, the right side of their back shaved, and the exposed skin wiped with 70% ethanol. One sponge disc per animal was aseptically implanted into a subcutaneous pouch, which had been made with curved artery forceps through a 1 cm long dorsal mid-line incision 4 cm away from the implant location (Fig 1A and 1B). Placement of sponge discs in this manner reduces the confounding influence of the wound incision over the implant. The incisions were closed with a silk braided non-absorbable suture. In this series of experiments, the implants of twenty-four mice were removed at day 1 or at day 10 post-implantation to analyze the inflammatory markers of both acute and chronic inflammatory processes (myeloperoxidase-MPO, n-acetyl-β-D-glucosaminidase- NAG, VEGF and TNF-α) and for cell content determination by flow cytometry. The schematic diagram (Fig 1C) shows the timeline of sponge implantation and removal.

### Enzymatic digestion of sponge implants and flow cytometry

Three sponge implants from each group (24 h and 10 days post-implantation) were minced with scissors in 1 ml HBSS, then 2.5 ml of filter-sterilized collagenase type 1 (Sigma Chemicals, St Louis, MO, USA) was added to the fragments. After incubation for 30 min at 37°C, the cells were centrifuged (500 g for 10 min at 4°C) and tested for viability. Cells isolated from sponges were suspended in Staining buffer (2% BSA in PBS) and incubated for 15 minutes at 4°C with Fc blocking (monoclonal Anti-CD16/32) (Biosciences, San Diego, CA, USA). Subsequently, cells were washed with FACS buffer, stained for surface molecules for 20 minutes at 4°C, suspended and maintained in 200 μl of PBS 2% paraformaldehyde (Sigma Aldrich, St. Louis, MO, USA). At least 50,000-gated events were acquired for analysis using FACSCanto-II (BD Biosciences, San Jose, CA, USA). The monoclonal antibody panels included the following antibodies: Rat anti-mouse F4/80 (clone BM8 –Cat.123123)-Pacific Blue (1:100), rat anti-mouse CD11b (clone M1/70 –Cat.101215)-PE/Cy7 (1:2000); Hamster anti-mouse CD11c (clone N418–117323)-APC/Cy7 (1:400), purchased from Biolegend (San Diego, CA, USA); Rat anti-mouse IA/IE (MHC-II) (clone 2G9 –Cat.562009)-FITC (1:2000); Rat anti-mouse GR1 (clone RB6-8C5–552093)-Percp-Cy5.5 (1:3000); Rat anti-mouse CD3 (clone 17A2 –Cat.555275)-PE (1:200); Rat anti-mouse CD8 (clone 53–6.7–01049A)-APC (1:1:400) purchased from BD Biosciences, and Rat anti-mouse CD4 (clone GK1.5 –Cat.25004181)-PE/Cy7 (1:1:5000) and Rat anti-mouse F4/80 (clone BM8–124801)-PE (1:100), purchased from eBiosciences. Data were analyzed using FlowJo Version 9.7.5 (TreeStar, Carrum Downs, Australia). A forward scatter (FSC-A) and side scatter (SSC-A) gate were used to initially remove debris and capture leucocytes (A in S1 Fig). Cells were gated based on CD11b expression (B in S1 Fig) and then F4/80 Low versus GR1 to select monocytes (GR1+ and GR1Low) and neutrophils (GR1+ F4/80Neg) (C and D in S1 Fig). A more detailed analysis of monocyte subpopulations was done based on GR1 expression and herein designated as:inflammatory monocytes (F4/80+CD11b+GR1-High) and patrolling monocytes (F4/80+CD11b+GR1-Low) (C in S1 Fig). F4/80 High GR1Low/Neg gate was used to characterize macrophages (D in S1 Fig). CD11c+ versus IA/IE–High (CD11C+IA/IEHi) gate was performed to characterize dendritic cells (E in S1 Fig). T lymphocytes were gated on CD3+ cells (F in S1 Fig).

### Tumor cell line and culture conditions

The tumor cell line 4T1 (tumor mammary gland) is a thioguanine-resistant variant that was selected from 410.4 without mutagen treatment [19] and obtained from the ATCC (American Type Culture Collection, Manassas, VA). Cells were maintained in RPMI 1640 medium (Hyclone, Logan, UT) containing 10% FBS, at 37°C in a humidified atmosphere of 5% CO_2_. Once confluent, the monolayer was harvested by incubation for 2 min with trypsin/EDTA; ethylenediaminetetra-acetic acid (0.025% and 0.02 w/v, respectively). The dislodged cells were centrifuged for 10 min and adjusted to the appropriate concentration in saline; 100 μl of the cell suspension (1x10^6^ cells) was injected into the right posterior flank of syngeneic mice in three different protocols. In one group, the tumor cells were inoculated in the subcutaneous space without the implant (tumor alone). In the other two groups, the tumor cells were inoculated in implants placed subcutaneously 24 h earlier (acute inflammation) or in implants placed 10 days earlier (chronic inflammation) Tumors were measured in two dimensions using a Vernier caliper and tumor volumes were calculated from the formula: TV (mm^3^) = 0.52 AB^2^, where A is the minor axis and B is the major axis [20].

### Removal of tumor alone and co-implanted in sponge matrix implant and tumor harvest

Fifteen days after tumor cell inoculation, the animals were anesthetized with ketamine and xylazine and later killed by cervical dislocation after removal of tumors, implants bearing tumors, implants alone, and blood sampling. The tissues were carefully dissected from the adherent tissue, removed, and weighed. They were then processed as described below and subjected to several assays.

In a series of experiments performed to examine the influence of the acute or chronic inflammation on tumor development, tumors alone and implant bearing-tumors were removed 15 days after inoculation of 4T1 cells, which were injected either in 24 hour-implants or in 10-day old implants. Two different groups of animals received sponge implant (without tumor inoculation), which were removed either at day 15 or 25 post-implantation. Table 1 shows the design protocol for this series of experiments.

**Table 1:** Experimental design protocol showing the groups, treatment regimen of cell inoculation and removal of tumors.

Experimental Groups	Day 0	Day 1	Day 10	Day 15	Day 25
**4T1**	Tumor cell injection			Tumor removal	
**AI**	Sponge implantation	Tumor cell injection **+** acute inflammation		Implant bearing tumor removal	
**CI**	Sponge implantation		Tumor cell injection **+** chronic inflammation		Implant bearing tumor removal

### Tissue extraction and hemoglobin (Hb) measurement

The extent of vascularization in the tumor samples (alone and implant-bearing tumors) was assessed by the amount of hemoglobin (Hb) detected in the tissue using the Drabkin method [21]. The tissues were homogenized Ultra–Turrax, (Schlappmuhler, Usingen, Germany) in 5 ml of Drabkin reagent (Labtest, São Paulo, Brazil) and centrifuged at 7000 *g* for 40 min. The supernatants were filtered through a 0.22-μm Millipore filter (Danvers, MA, USA). The hemoglobin concentration in the samples was determined spectrophotometrically by measuring absorbance at 540 nm using an ELISA plate reader and compared against a standard hemoglobin curve. The hemoglobin content in the implant was expressed as μg Hb per mg wet tissue.

### Determination of myeloperoxidase and N-acetylglucosaminidase activities

The number of neutrophils in the samples was measured by assaying myeloperoxidase (MPO) activity as previously described [22–24]. The tissues were homogenized in pH 4.7 buffer (0.1 M NaCl, 0.02 M NaPO4, 0.015 M NaEDTA), and centrifuged at 200 g for 10 min. The pellets were then re-suspended in 0.05 M NaPO4 buffer (pH 5.4) containing 0.5% hexadecyltrimethylammonium bromide (HTAB) followed by three freeze-thaw cycles using liquid nitrogen. MPO activity in the supernatant samples was assayed by measuring the change in absorbance (optical density; OD) at 450 nm using tetramethylbenzidine (1.6 mM) and H_2_O_2_ (0.3 mM). The reaction was terminated by adding 50 μl of H2SO4 (4M). Results were expressed as a change in OD per g of wet tissue.

The infiltration of mononuclear cells was quantified by measuring the levels of the lysosomal enzyme N-acetylglucosaminidase (NAG) present in high levels in activated macrophages [22–24]. The samples were homogenized in NaCl solution (0.9% w/v) containing 0.1% v/v Triton X-100 (Promega, Madison, WI, USA) and centrifuged (200 g; 10 min at 4°C). Samples (100 μl) of the resulting supernatant were incubated for 10 min with 100 μl of p-nitrophenyl-N-acetyl-beta-D-glucosaminide (Sigma-Aldrich, St Louis, MO, USA) prepared in citrate/phosphate buffer (0.1 M citric acid, 0.1 M Na_2_HPO_4_; pH4.5) to yield a final concentration of 2.24mM. The reaction was terminated by adding 100μl of 0.2 M glycine buffer (pH 10.6). Hydrolysis of the substrate was determined by measuring the absorption at 400 nm. Results were expressed as nmol/mg wet tissue.

### Measurement of VEGF, TNF-α, and CCL2 levels

The measurement of relevant pro-angiogenic and pro-inflammatory cytokines (VEGF, TNF-α, CCL2) was carried out in the supernatant (50 μl) of homogenized tissue samples and in the serum (20 μl) from tumor-bearing and non-tumor-bearing animals, using Immunoassay Kits (R&D Systems, Minneapolis, USA) and following the manufacturer’s protocol. Briefly, dilutions of cell-free supernatants were added in duplicate to ELISA plates coated with a specific murine monoclonal antibody against cytokine, followed by adding a second horseradish peroxidase-conjugated polyclonal antibody, also against cytokine. After washing to remove any unbound antibody-enzyme reagent, a substrate solution (50μL of a 1:1 solution of hydrogen peroxide and tetramethylbenzidine 10mg/ml in DMSO) was added to the wells. Color development was terminated after 20 min incubation with 2N sulfuric acid (50 μL) and color intensity was measured at 540 nm on a spectrophotometer (E max–Molecular Devices). Standards were 0.5-log10 dilutions of recombinant murine cytokines from 7.5 pg ml-1 to 1000 pg ml-1 (100 μl). The results were expressed as pg cytokine per mg wet tissue or per mL.

### Histological staining

The sponge implants, tumors, and sponge implant bearing tumors were fixed in 10% buffered formalin, pH 7.4, and processed for paraffin embedding. Sections with a thickness of 5 mm were stained with hematoxylin/eosin (H&E) for light microscopic studies. The images were captured with a planapochromatic objective (40x) in light microscopy (final magnification = 400x). The images were digitized through a JVC TK-1270/JCB microcamera and transferred to an analyzer (software Image-Pro Plus 4.5, Media Cybernetics, Inc. Silver Spring, MD, USA).

### Statistical analysis

Results are presented as mean±SEM. The assumptions of normality and homoscedasticity were determined for subsequent statistical analysis. Comparisons among the groups were made using one-way analysis of variance (ANOVA) followed by the Newman–Keuls correction factor for multiple comparisons as a post-test. Differences between means were considered significant when P values were <0.05. Analysis of the data was carried out using the GraphPad Prism program 6.0 version (La Jolla, CA, USA).

## Results

Subcutaneous implantation of polyether-polyurethane sponge discs in mice has consistently been shown to induce an inflammatory reaction that progresses from acute to chronic processes over time [22, 23]. We used this experimental system to analyze the influence of acute or chronic inflammation on the development of mammary tumors (4T1 cells). The inflammatory exudate from the implants removed twenty-four hours after implantation was composed of higher levels of myeloperoxidase (MPO) compared with those from 10-day old implants. Conversely, the levels of NAG were higher in older implants than those implanted for 24 h. As these two inflammatory enzymes are produced mainly by activated neutrophils (MPO) and macrophages (NAG), it is concluded that, at 24 h Post-implantation, an acute inflammatory phase is predominant in the implant compartment, whereas at day 10, a more chronic phase is predominant. To determine whether the pro angiogenic (VEGF) and pro inflammatory (TNF-α) cytokines were involved in both phases of the inflammatory process induced by the synthetic matrix; their levels were measured in the supernatant. The amount of the cytokines was similar at both time points (Table 2).

**Table 2:** Inflammatory markers in free-tumor sponge 24 h and 10 days post-implantation. MPO-Myeloperoxidase; NAG- n-acetyl-β-D-glucosaminidase; All results are the mean±sem; (n = number of animals);

Time after implant	MPO activity (OD/mg tissue)	NAG activity (nmol/mg tissue)	VEGF levels (pg/mg tissue	TNF-α levels (pg/mg tissue)
24 h	6.4±0.8 (n = 6) *	1.02±0.14 (n = 3) *	0.6±0.15 (n = 4)	0.25±0.06 (n = 3)
10 days	2.9±0.5 (n = 3)	1.55±0.12 (n = 3)	0.5±0.18 (n = 4)	0.18±0.002 (n = 3)

*p< 0.*05 24 h compared with 10 days (T-test)*.

**Fig 2:**
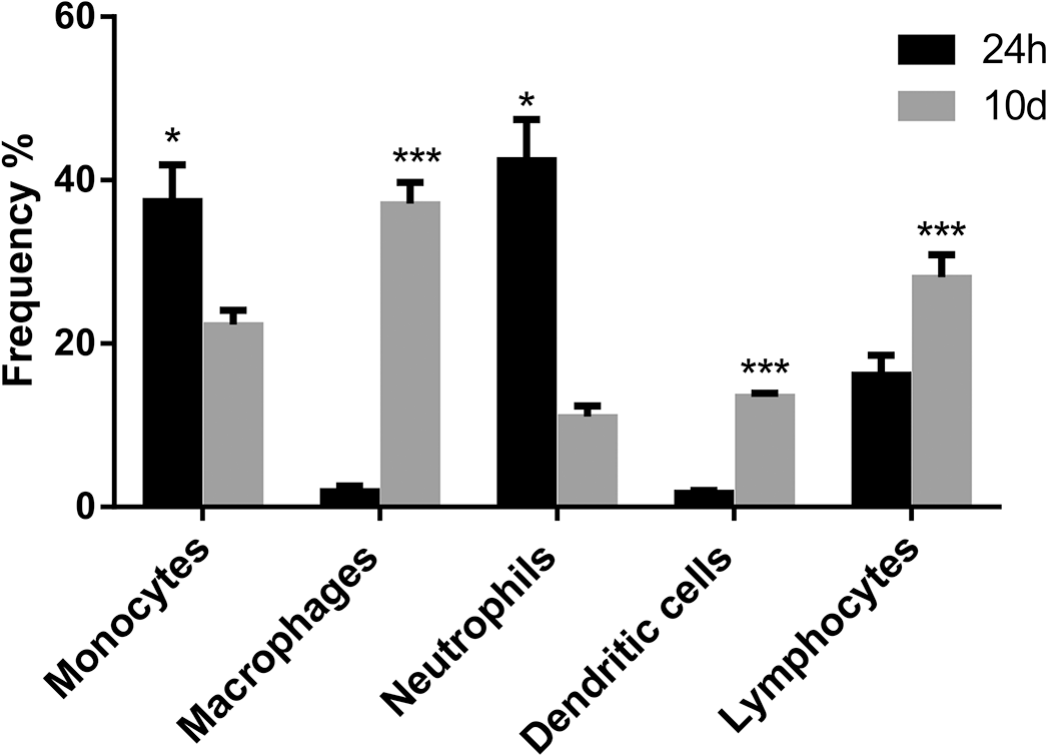
Cytometry analysis of leukocytes isolated from implants. Frequency of leukocytes isolated from sponges after 24 h or 10 days of implantation. Analysis of the frequency of cells comparing 24 hour x 10 days for shows predominance of neutrophils at 24 h post-implantation versus predominance of monocytes/macrophages at 10 days post-implantation.

Thus, it is concluded that they are components of the implant environment during both acute and chronic inflammation. More direct evidence of the cell types present in the implant compartment at both time intervals in which the tumor cells were inoculated was characterized by flow cytometry after 24 hours or 10 days of sponge implantation. Flow cytometry analysis has shown differences in cell type population when comparing 24 hours and 10 days. After 24 hours, the leukocyte population was characterized mainly by neutrophils (42.53% +/- 8.45) and monocytes (37.53% +/- 7.48), with some lymphocytes (16.27% +/- 4.0) and few dendritic cells (1.82% +/- 0.36). A few natural killer cells could be seen in a very low frequency, less than 0.5% (data not shown). At 10 days, the leukocyte population was characterized mainly by macrophages (37.10% +/- 4.54), lymphocytes (28.1% +/- 4.77), and monocytes (22.33% +/- 3.05), with some dendritic cells (13.60% +/- 0.55) and neutrophils (11.07% +/- 2.27). Comparing the leukocyte population between the two time points, it was possible to see a higher number of monocytes and neutrophils in 24 hours, when compared to 10 days, and very few macrophages were detectable after 24 hours of sponge implant. After 10 days, the frequency of macrophages had increased considerably, as had the frequency of dendritic cells and lymphocytes (Fig 2).

**Fig 3:**
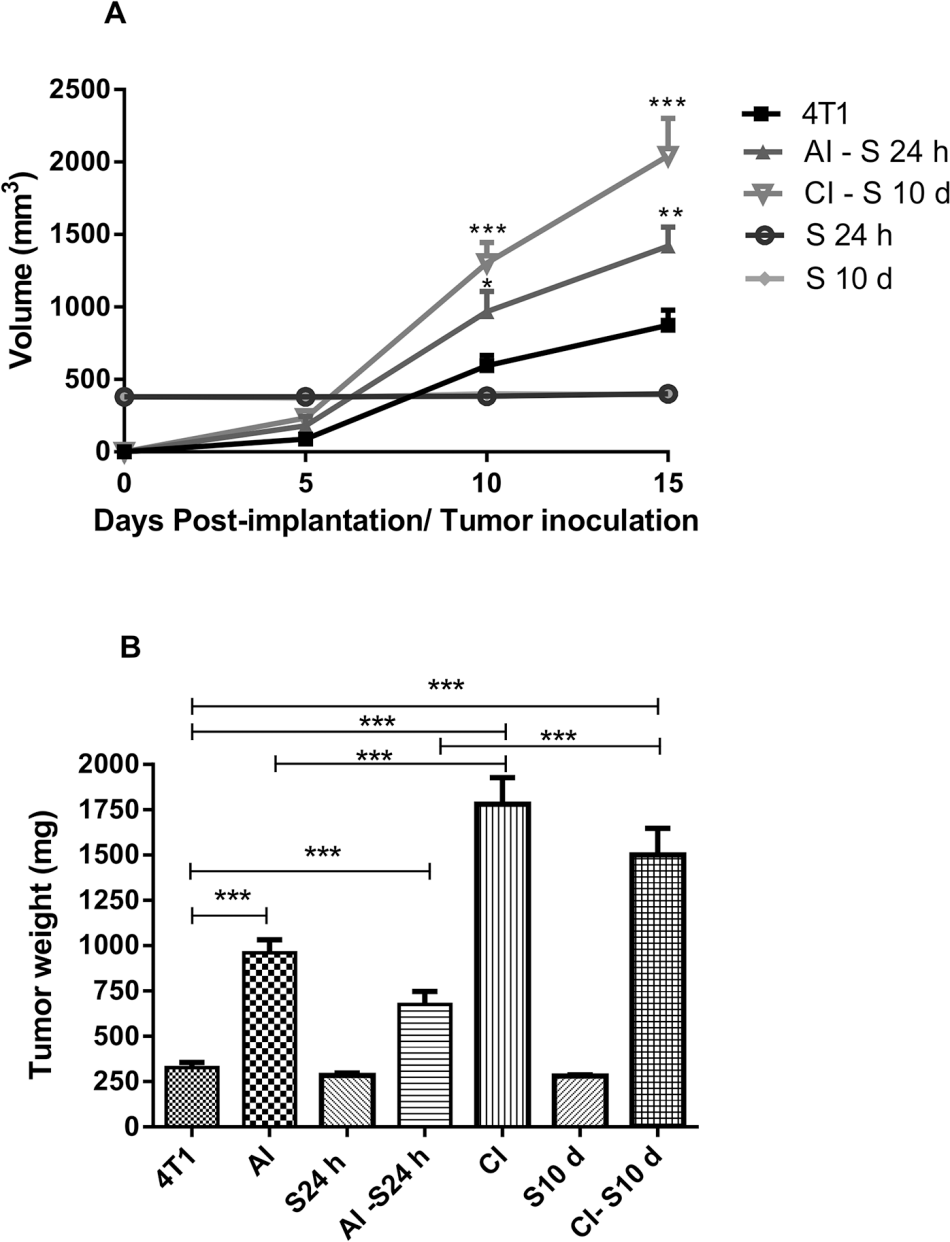
Tumor and implant measurement. Kinetics of tumor growth in A (volume), and tumor and implant wet weight (mg) in B. The sponge volume and wet weight were subtracted from the total volume/weight of sponge bearing tumors (AI-S 24h and CI–S10 d). In the chronic inflammation tumors values were different from 4T1 alone. In the acute inflammation tumors, the volume/weight were also different compared with tumor volume alone. Values are mean±sem of 10 animals in each group. *p<0.05; **p<0.01; ***p<0.001 compared with tumor alone (4T1) or AI and CI–ANOVA. AI (acute inflammation + 4T1); CI (chronic inflammation + 4T1).

In these two distinct inflammatory environments, inoculation of 4T1 tumor cells yielded tumors that grew differentially over a 15-day period. In situ examination showed that the tumor mass was more prominent in sponge implant bearing tumors compared with tumor alone (A-D in S2 Fig). 4T1 tumor volume (mm^3^) measured in different time points was significantly less compared with the volumes of sponge implant bearing tumors in acute and chronic inflammation. The sponge volume was subtracted from the total volume of sponge bearing tumors. There was no variation in sponge volume during the experimental period (Fig 3A). The wet weight (mg) of tumors alone was 327.2±28.8 versus 960±71.6 (4T1+Acute Inflammation; AI), versus 1781±147 (4T1+Chronic Inflammation; CI) Fig 3B. By subtracting the sponge weight from the acute or chronic inflammation tumors the values were 676.6±71.64 (4T1+Acute Inflammation–sponge 24 h; AI–S 24 h), versus 1500 ± 146.7 (4T1+Chronic Inflammation–sponge 10 d; CI–S 10 d), still different from 4T1 alone. The wet weight of the sponge implants remained unchanged for the whole experimental period (Fig 3B). Histological analysis of the fibrovascular tissue that infiltrated the sponge matrix showed that it was composed of microvessels, fibroblasts, inflammatory infiltrate consisting of neutrophils, macrophages, and a collagen rich extracellular matrix (A in S3 Fig). Sections of the tumors with or without the support of the synthetic matrix inflammatory presented neoplastic cells with nuclei of increased dimension and pale eosinophilic cytoplasm with indistinct cell borders, high cellular pleomorphism, and necrotic areas in the compact tumor mass. (B-D in S3 Fig).

**Fig 4:**
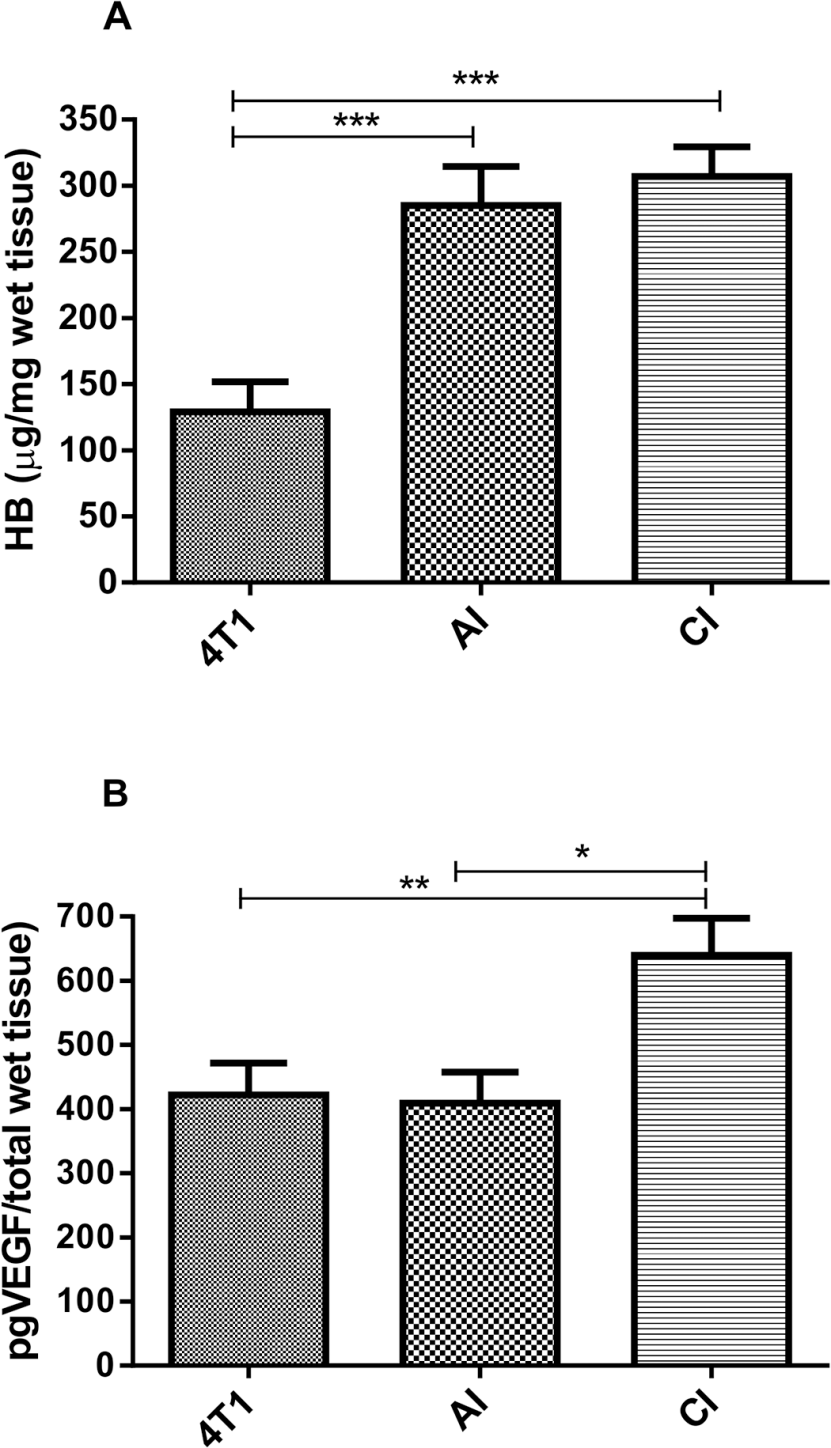
Effects of acute (AI) or chronic inflammation (CI) on angiogenesis of 4T1 mammary tumor. (A) hemoglobin content-Hb and (B) VEGF levels. Inoculation of 1x10^6^ tumor cells in subcutaneous space, intraimplant (24h or 10 days post-implantation) induced more angiogenesis in implant-bearing tumors. Values are mean±sem of 10 animals in each group. *p<0.05; **p<0.01; ***p<0.001 –ANOVA. AI (acute inflammation + 4T1); CI (chronic inflammation + 4T1).

Next, we characterized the tumors’ vascularization index by assaying the hemoglobin content. In implant-bearing tumors, the amount of Hb was higher than that of the tumors alone. The amount of Hb did not vary when 4T1 tumor cells were grown in an acute or chronic inflammatory environment (Fig 4A). The levels of pro-angiogenic cytokine (VEGF) were higher in implant-bearing tumors when 4T1 cells were grown in 10-day old implants as compared to the other two groups (Fig 4B). This result is fully compatible with the well-established role of VEGF in tumor development

**Fig 5:**
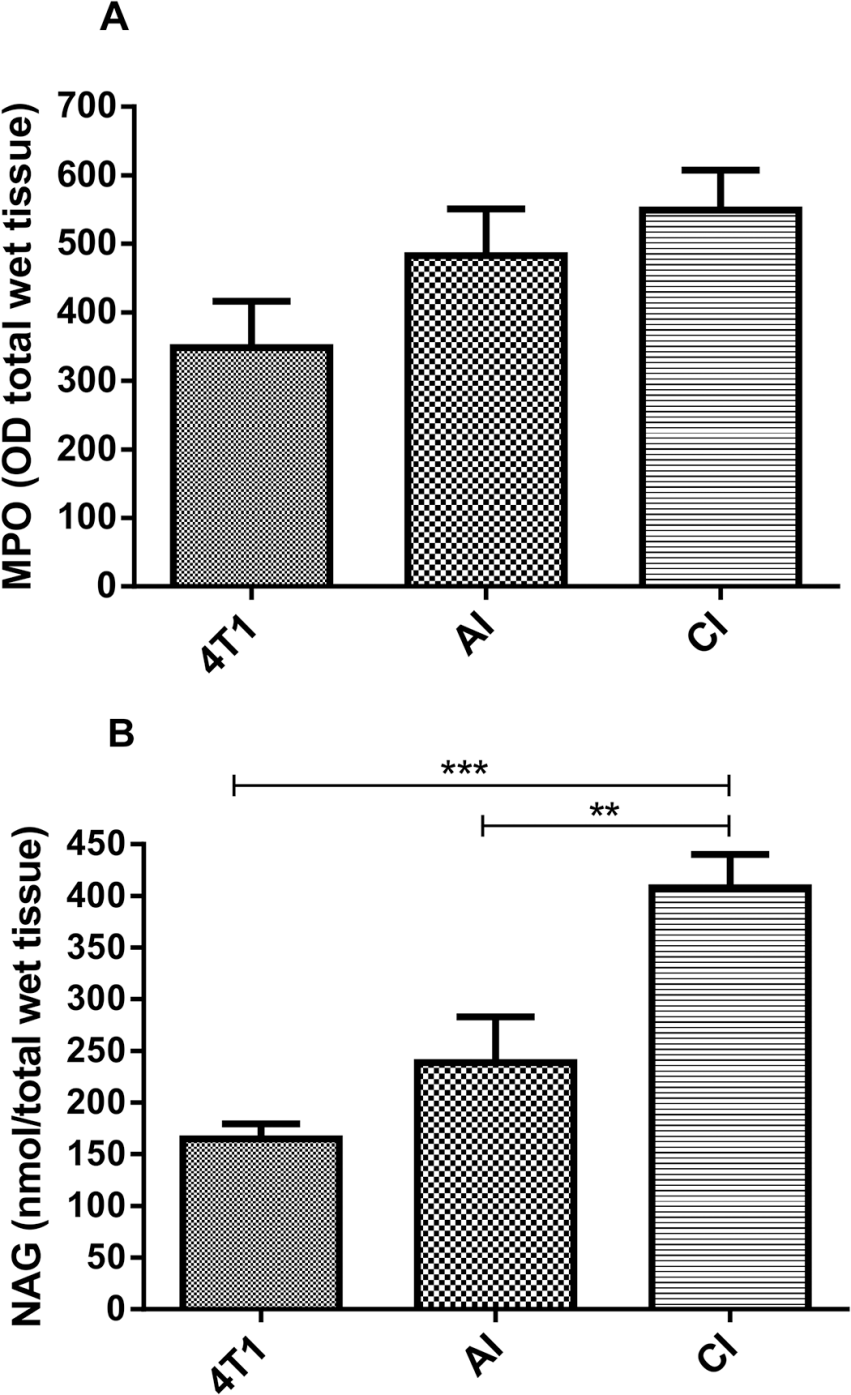
Effects of acute (AI) or chronic inflammation (CI) on inflammatory enzyme activities (A) myeloperoxidase-MPO, (B) n-acetyl-β-D-glucosaminidase of 4T1 mammary tumor. NAG activity was higher in implant-bearing tumors. Values are mean±sem of 10 animals in each group. **p<0.01; ***p<0.001- ANOVA. AI (acute inflammation + 4T1); CI (chronic inflammation + 4T1).

Overall, the levels of the inflammatory markers evaluated (NAG, TNF-α, CCL2) in the implant-bearing tumors were also influenced by the inflammation phase in which the tumor cells were grown. There was no difference in MPO activity among the groups (Fig 5A), but NAG activity was higher in tumor-bearing implants in both groups as compared with tumor alone (Fig 5B).

**Fig 6:**
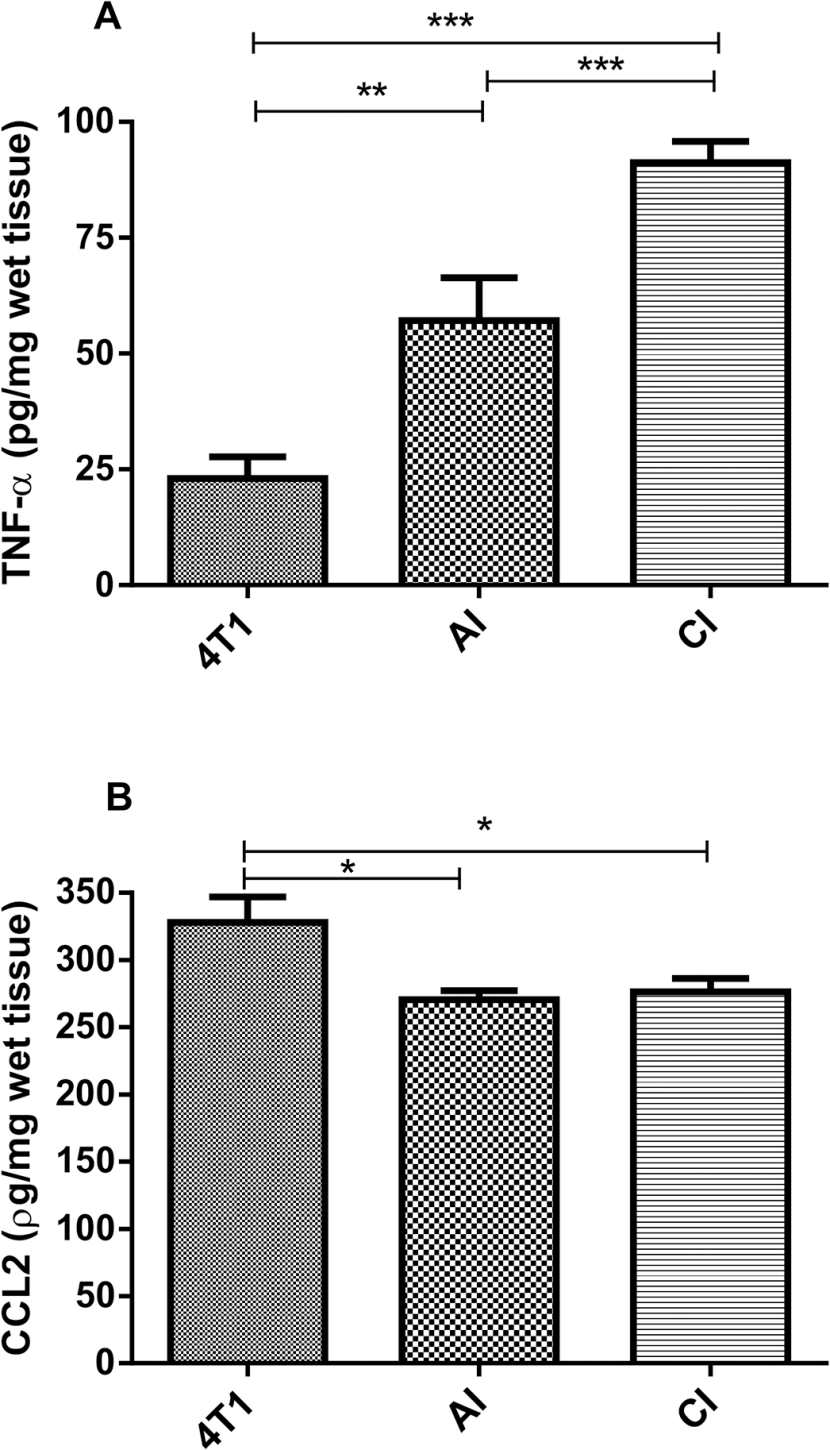
Effects of acute (AI) or chronic inflammation (CI) on cytokine production of 4T1 mammary tumor. (A) TNF-α; (B) CCL2. TNF-α levels were higher in implant-bearing tumors. Values are mean±sem of 10 animals in each group. *p<0.05**; p<0.01; ***p<0.001 –ANOVA. AI (acute inflammation + 4T1); CI (chronic inflammation + 4T1).

The levels of TNF-α were also higher in both groups of implant-bearing tumors than those in the tumor alone (Fig 6A). Conversely, the levels of CCL2 chemokine were lower in implant-bearing tumors from both groups, as compared with those from tumor alone (Fig 6B).

**Fig 7:**
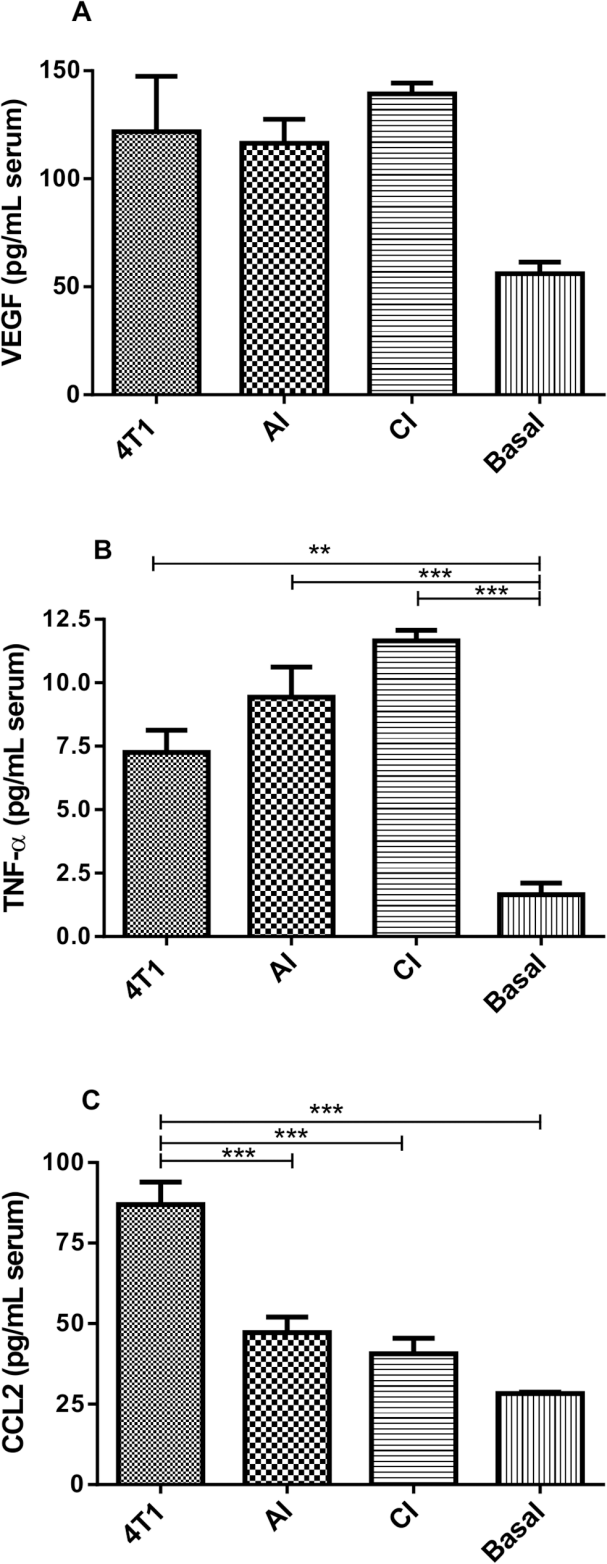
Systemic levels of VEGF (A) and TNF-α (B) in tumor-bearing mice, CCL2 (C). VEGF levels were similar in tumor-bearing animals, TNF-α levels were higher in serum from tumor-bearing animals. CCL2 levels were higher in serum from implant-free tumor-bearing animals. Values are means±sem of 10 animals in each group. *p<0.05; **p<0.01; ***p<0.001 –ANOVA. AI (acute inflammation + 4T1); CI (chronic inflammation + 4T1).

We also analyzed cytokine levels (VEGF, TNF- and CCL2) in serum from mice-bearing tumors and non-bearing tumors. The systemic levels of VEGF were similar in the animals bearing tumors (Fig 7A). However, the circulating levels of TNF-α were higher in implant-bearing tumor animals than in those of the animals bearing tumor alone (Fig 7B). Conversely, systemic levels of CCL2 were higher in animals bearing tumor alone than those in which the tumor cells grew intra-implant (Fig 7C). It is clear that the inflammatory component of the implant compartment differentially modulated the production of cytokines, locally and systemically.

## Discussion

It has become evident that inflammation initiates and/or exacerbates the development of 15 to 20% of all malignancies [2, 25, 26]. This notion is supported by the fact that the inflammatory tumor microenvironment is characterized by the presence of inflammatory cells (macrophages, neutrophils, lymphocytes, eosinophils, and mast cells) and by a number of independent studies showing that many malignancies arise from areas of infection and inflammation [1, 2, 4, 27]. The association between inflammation and tumorigenesis has also been demonstrated using experimental strategies in which distinct tumor cell lines and different populations of inflammatory cells are co-cultivated in *in vitro* and/or *in vivo* systems. There is, however, a lack of information regarding the influence of acute versus chronic inflamed environments on the development of solid tumors and whether both distinct inflammatory processes alter the pattern of key components of tumor tissue (angiogenesis, inflammatory cell recruitment/activation, and cytokine production). The chosen experimental tumor model, the mouse mammary carcinoma 4T1, originally isolated as subpopulation 410.4 derived from a spontaneous mammary tumor in Balb/c mice, presents mixed myeloid cell infiltrates, produces various pro-inflammatory cytokines, and is considered a good breast cancer model [28, 29]. Using this combination of tumor cell and inflammatory environment, we have shown that inoculation of 4T1 tumor cells (1x10^6^) in a subcutaneous polyether-polyurethane sponge matrix, implanted for 24h or 10 days, induced the development of mammary tumors that differed from that in which the tumor cells were inoculated in the animals’ subcutaneous space. We observed that subcutaneous implantation of a polyether polyurethane matrix induced the infiltration of leukocytes at both evaluated time points and the products of their activities (inflammatory enzymes and cytokines) confirmed our previous findings [22,23,30]. Evaluation by flow cytometry of the inflammatory cell profile from 24h-implants, characterized herein for the first time, showed that the predominant cell types were neutrophils (42.53% +/- 8.45) and monocytes (37.53% +/- 7.48), whereas in 10-day old implants the population of leukocytes was characterized mainly by macrophages (37.10% +/- 4.54), lymphocytes (28.1% +/- 4.77), and monocytes (22.33% +/- 3.05). Both distinct profiles are characteristic of acute and chronic inflammation, respectively, and clearly contributed to the differential growth of the mammary tumor. Thus, most of the parameters evaluated (tumor growth, NAG activity, VEGF, TNF-α production) were higher in inflammation-promoted tumor groups when compared with tumors grown in the subcutaneous space without additional inflammatory support from the sponge. Our findings are consistent with previous reports showing that implantation of tumor cells in inflamed environments potentiates and/or modifies some features of malignant cells. For instance, when QR32 tumor cells (a poorly tumorigenic clone) were co-implanted with a gelatin sponge, they acquired enhanced tumorigenicity and metastatic ability [13,18]. Initiation and promotion of sarcoma development have also shown to be associated with foreign bodies [14,31]. Increased expression of inducible NOS (an inflammatory marker) in melanoma-bearing implants or in colon 26-bearing-implants compared with non-tumor-bearing implants has also been shown, implying further association between tumorigenesis and inflammation [16]. The fact that the tumor burden was 2-fold when 4T1 cells were inoculated in 10-day old implants compared with the size of tumors grown in the acute inflammatory environment was, to some extent, expected considering that the tumor cells were hosted in a highly vascularized bed (fibrovascular proliferative tissue induced by the synthetic matrix). These findings are in agreement with the well-established importance of angiogenesis to tumor development [32,33]. It was interesting to find out that the levels of pro-angiogenic cytokine, VEGF, and those of TNF-α were distinctly influenced by both inflammatory processes. Thus, VEGF and TNF-α in the tumor mass were more pronounced when 4T1 cells were inoculated in 10-day old implants when compared with the same features of tumors grown in 24h implants. In turn, tumors grown in the subcutaneous space without the additional inflammatory support from the sponge were the smallest and produced less cytokines, except for CCL2. Our findings are in agreement with the notion of the contribution of neutrophils to tumorigenesis as shown earlier by Weitzman et al. 1985 [34]. In their study, C3H mouse fibroblast cell line was converted to form tumors in nude mice after co-culture with neutrophils. Others have confirmed the role of neutrophils in tumorigenic conversion and the transformation of benign tumor cells into malignant metastatic phenotype [13, 35, 36].

Interestingly, tumor alone produced more CCL2, locally and systemically. It has been reported that the production of CCL2, constitutively or in response to inflammatory stimuli by 4T1 cells, is responsible for the initiation and progression of 4T1 tumors [10]. However, the production of VEGF and TNF-α systemically was more pronounced when the 4T1 cells were hosted in the implants. It is possible that both tumor cells and inflammatory infiltrate acted synergistically, producing the cytokines that, in turn, amplified neoplastic proliferation. In fact, both VEGF and TNF-α are part of a repertoire of molecules responsible for a number of events involved in tumor progression (angiogenesis, macrophage recruitment, proliferation, and metastasis) [37].

The cellular profile of the murine mammary carcinoma is composed of mixed myeloid cell infiltrates consisting of a progressive increase of CD45+ hematopoietic cells and CD11+ myeloid cells [38, 39]. Whether the distinct inflammatory environments of the implants (acute or chronic inflammation) altered this profile has not been determined. Future experiments may address this issue by examining the phenotype of 4T1 cells exposed to a distinct population of inflammatory cells.

In conclusion, our experiments were intended to comparatively assess the influence of acute and chronic inflammation on the development of the murine mammary tumor and whether these two processes would alter angiogenesis, inflammation, and cytokine production, locally and systemically. It is clear from our results that both inflammatory processes potentiated the development of 4T1 tumor. However, chronic inflammation was more effective than acute inflammation in inducing VEGF and TNF-α and tumor progression. These inflammation-related differences in tumor growth may provide new insights into the contribution of different inflammatory cell populations to cancer development.

## Supporting Information

S1 Data Analyses of sponge implants alone, 1 and 10 days after inoculation of 4T1 tumors. Data set from the statistical analyses of all experiments.
(PDF)

S1 Fig Flow cytometry strategy to define the myeloid and lymphoid populations isolated from sponge implants. (A) FSC-A x SSC-A profile from total events acquired showing the gate used to eliminate debris. (B) Histogram defining the CD11b+ myeloid population. (C and D) Dot plots to define monocytes (F4/80lo), macrophages (F4/80Hi) and neutrophils (F4/80Neg). (E) Dot plot showing the profile of dendritic cells based on CD11c+ and MHC-IIHi. (F) T cell population based on CD3+ cells.
(TIF)

S2 Fig Representative macroscopic images of sponge implant and tumors *in situ* and tumor cell inoculation in sponge implants. In A, sponge implant, in B, tumor alone; in C, tumor cells were inoculated in a 24 h-implant; in D, tumor cell were inoculated in a 10-day old implant.
(TIF)

S3 Fig Representative histological sections stained with H&E of fibrovascular tissue in sponge implant (A) and tumor tissue (B-D) The pores of the sponge matrix, seen as triangular shapes, are infiltrated by a fibrovascular tissue composed of microvessels fibroblasts, inflammatory infiltrate consisting of neutrophils, macrophages, and a collagen rich extracellular matrix. In tumor sections with or without the support of the synthetic matrix, neoplastic cells occupies the implant compartment along with blood vessels, inflammatory cells and stroma (B-D). Scale bar, 50 μm; * matrix; arrows, tumor cells.
(TIF)
