# Correction: Differential Contribution of Acute and Chronic Inflammation to the Development of Murine Mammary 4T1 Tumors

**DOI:** 10.1371/journal.pone.0138408

**Published:** 2015-09-14

**Authors:** Celso Tarso Rodrigues Viana, Pollyana Ribeiro Castro, Suzane Motta Marques, Miriam Teresa Paz Lopes, Ricardo Gonçalves, Paula Peixoto Campos, Silvia Passos Andrade

The following information is missing from the Funding section: This study was supported by Fundação de Amparo à Pesquisa do Estado de Minas Gerais (FAPEMIG).
