# Supplementary material for: Differential Contribution of Acute and Chronic Inflammation to the Development of Murine Mammary 4T1 Tumors
Source: PLoS One. 2015 Jul 9;10(7):e0130809. doi: 10.1371/journal.pone.0130809 (PMC4497676; doi:10.1371/journal.pone.0130809)
Supplement: S1 Data — Data set from the statistical analyses of all experiments. (PDF) [file pone.0130809.s001.pdf]

Flow cytometry of leukocytes Data (mean±SEM)					
Days post sponge implantation	Monocytes	Macrophages	Neutrophils	Dendritic cells	Lymphocytes
1 d (24 h)	37.53±4.321	2.3±0.5	42.53±4.9	1.827±0.2128	16.27±2.314
10 d	22.33±1.761	37.10±2.621	11.07±1.31	13.60±0.3215	28.10±2.759

Days post sponge implantation s	24h			10d		
**Flow cytometry of leukocytes Data**	Cell Frequency %					
Monocytes	41.50	42.20	28.90	25.40	22.30	19.30
Macrophages	1.00	2.00	3.00	38.60	32.00	40.70
Neutrophils	34.00	42.70	50.90	9.00	13.50	10.70
Dendritic cells	2.11	1.96	1.41	14.10	13.00	13.70
Lymphocytes	19.20	17.90	11.70	29.10	22.90	32.30

**4T1 mammary tumor growth (Volume mm^3) mean of 10 animals**				**Volume mm^3 sponge implant Mean 10**		**4T1 mammary tumor growth (Volume mm^3) mean of 10 animals**	
	**4T1**	**AI**	**CI**	**S 24 h**	**S 10 d**	**AI- Mean S24h**	**CI- Mean S10d**
5 Days	89.14	538.35	606.725	380	372	158.35	234.5
10 Days	594.886	1307.00	1715.13	390.1	400	917.1	1315
15 Days	874.498	2008.03	2422.91	400	395	1420	2027.9

**4T1 mammary tumor growth mass (wet weight/mg) mean of 10 animals**		
**4T1**	**AI**	**CI**
327.2± 28.76	960.9± 71.64	1781 ± 146.7
**4T1 mammary tumor growth mass (mg) of 10 animals**		
**4T1**	**AI**	**CI**
326.5	931.0	1656.4
420.2	1231.7	1794.1
266.2	1221.0	1834.9
297.9	832.8	1401.2
533.7	775.5	1431.2
227.2	925.9	1290.4
237.1	782.6	2812.3
307.0	1362.7	2274.0
332.0	818.2	1851.8
324.4	727.7	1465.8

**Sponge implant mass (mg) mean of 10 animals**	
**Sponge 24h**	**Sponge 10 days**
284.325	281.1667

**AI**	**Sponge 24h**	**mean S24h**	**AI- mean S24h**	**CI**	**Sponge 10d**	**Mean S 10d**	**CI - Mean S 10d**
931	308.3	284.325	646.675	1656.4	292.7	281.17	1375.2

1231.7	323		947.375	1794.1	278.9		1512.9
1221	323.3		936.675	1834.9	264.9		1553.7
832.8	251.2		548.475	1401.2	278.5		1120.0
775.5	238.9		491.175	1431.2	269.4		1150.0
925.9	249.8		641.575	1290.4	302.6		1009.2
782.6	320.3		498.275	2812.3	292.7		2531.1
1362.7	259.8		1078.375	2274	278.9		1992.8
818.2			533.875	1851.8			1570.6

**4T1 mammary tumor and sponge weight (mg) mean ± SEM of 10 animals**							
	**4T1**	**AI**	**CI**	**Sponge 24h**	**Sponge 10 days**	**AI-S24h**	**CI - S 10d**
15 Days	327.2 ± 28.76	960.9 ± 71.64	1781 ± 146.7	284.3± 13.25	281.2± 5.79	676.6 ± 71.64	1500 ± 146.7

**Hb (mg/mg wet tissue)**		
**4T1**	**AI**	**CI**
75.80	295.60	262.13
154.07	462.70	343.39
317.46	352.96	268.22
95.86	289.55	199.90
142.38	184.47	259.23
111.15	261.59	281.56
66.35	168.63	358.78
81.27	223.25	401.68
116.10	386.28	422.75
129.09	227.33	273.35
**Hb (mg/mg wet tissue)) mean ± SEM of 10 animals**		
**4T1**	**AI**	**CI**
129.0±22.84	285.2±29.42	307.1±22.46

**VEGF (pg/mg wet tissue)**		
**4T1**	**AI**	**CI**
211.96	703.53	1101.16
369.04	462.72	775.28
373.42	595.95	534.06
284.58	289.55	567.45
535.50	186.00	560.45
321.10	452.51	567.35
664.44	304.73	480.95
640.87	399.85	624.17
518.16	319.78	672.21
301.78	376.84	514.16
**VEGF (pg/mg wet tissue) mean ± SEM of 10 animals**		
**4T1**	**AI**	**CI**
422.1±49.62	409.1±48.35	639.7±57.88

MPO activity (OD/g wet tissue)		
**4T1**	**AI**	**CI**
375.0	338.8	302.0
324.0	487.0	291.0
781.0	401.0	490.0
668.0	700.0	643.0
343.0	315.0	499.0
170.0	326.0	500.0
160.0	483.0	892.0
235.0	610.0	731.0
171.0	943.0	509.0
258.0	222.0	636.0
**MPO activity (OD/g wet tissue) mean ± SEM of 10 animals**		
**4T1**	**AI**	**CI**
348.5±67.53	482.6±68.58	549.3±58.19

NAG (nmol/ml-1/mg wet tissue)		
**4T1**	**AI**	**CI**
257.77	177.17	538.53
125.50	145.70	394.60
103.50	471.58	547.70
183.14	110.18	389.06
192.30	432.07	394.05
147.12	159.32	188.50
195.95	156.53	369.37
119.11	412.93	483.86
138.45	162.60	360.79
185.42	158.20	410.60
**NAG (nmol/ml-1/mg wet tissue) mean ± SEM of 10 animals**		
**4T1**	**AI**	**CI**
164.8±14.73	238.6±44.25	407.7±32.48
**TNF α (pg/mg wet tissue)**		
**4T1**	**AI**	**CI**
9.45	63.1930	66.68
49.82	82.6800	97.67
28.89	28.5300	98.26
27.19	77.3000	93.91
13.57	82.6800	89.96
9.87	28.5300	111.94
26.34	71.2100	95.44
18.96	23.0600	66.31
		89.78
		101.98
**TNF α (pg/mg wet tissue) mean ± SEM of 10 animals**		
**4T1**	**AI**	**CI**

23.01±4.71	57.15±9.20	91.19±4.58

**CCL 2 (pg/mg wet tissue)**		
**4T1**	**AI**	**CI**
302.08	262.30	272.28
342.15	277.75	268.84
244.62	294.11	254.48
321.47	291.06	255.23
365.40	239.23	250.19
369.36	287.03	294.43
371.10	232.33	288.57
362.0	285.87	250.5
398.09	262.12	355.00
236.28	273.65	273.94
**CCL 2 (pg/mg wet tissue) mean ± SEM of 10 animals**		
**4T1**	**AI**	**CI**
327.8±19.06	270.5±6.76	276.3±10.02

	**(pg/ml serum)**			
	**4T1**	**AI**	**CI**	**Basal**
**VEGF**	121.9±25.56	116.5±11.06	139.3±4.982	56.05±5.436
**TNFα**	7.257± 0.8707	9.436± 1.182	11.65± 0.4213	1.652 ±0.4538
**CCL- 2**	86.92 ±7.067	47.22 ±4.784	40.62± 4.868	28.25 ±0.4593
