# Supplementary figures and images for: Differential Contribution of Acute and Chronic Inflammation to the Development of Murine Mammary 4T1 Tumors
Source: PLoS One. 2015 Jul 9;10(7):e0130809. doi: 10.1371/journal.pone.0130809 (PMC4497676; doi:10.1371/journal.pone.0130809)

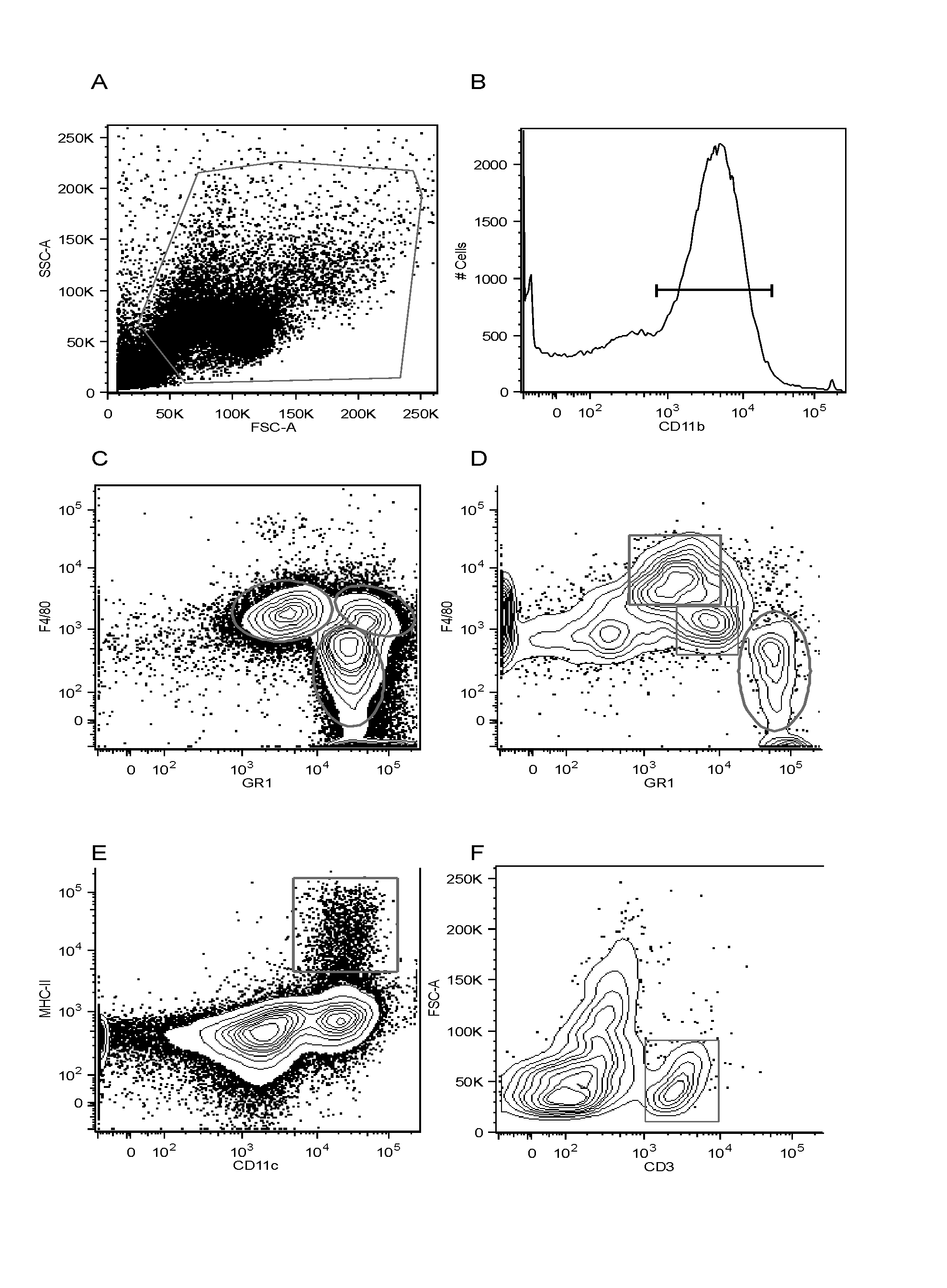

Supplement: S1 Fig — (A) FSC-A x SSC-A profile from total events acquired showing the gate used to eliminate debris. (B) Histogram defining the CD11b+ myeloid population. (C and D) Dot plots to define monocytes (F4/80lo), macrophages (F4/80Hi) and neutrophils (F4/80Neg). (E) Dot plot showing the profile of dendritic cells based on CD11c+ and MHC-IIHi. (F) T cell population based on CD3+ cells. (TIF) [file pone.0130809.s002.tif]

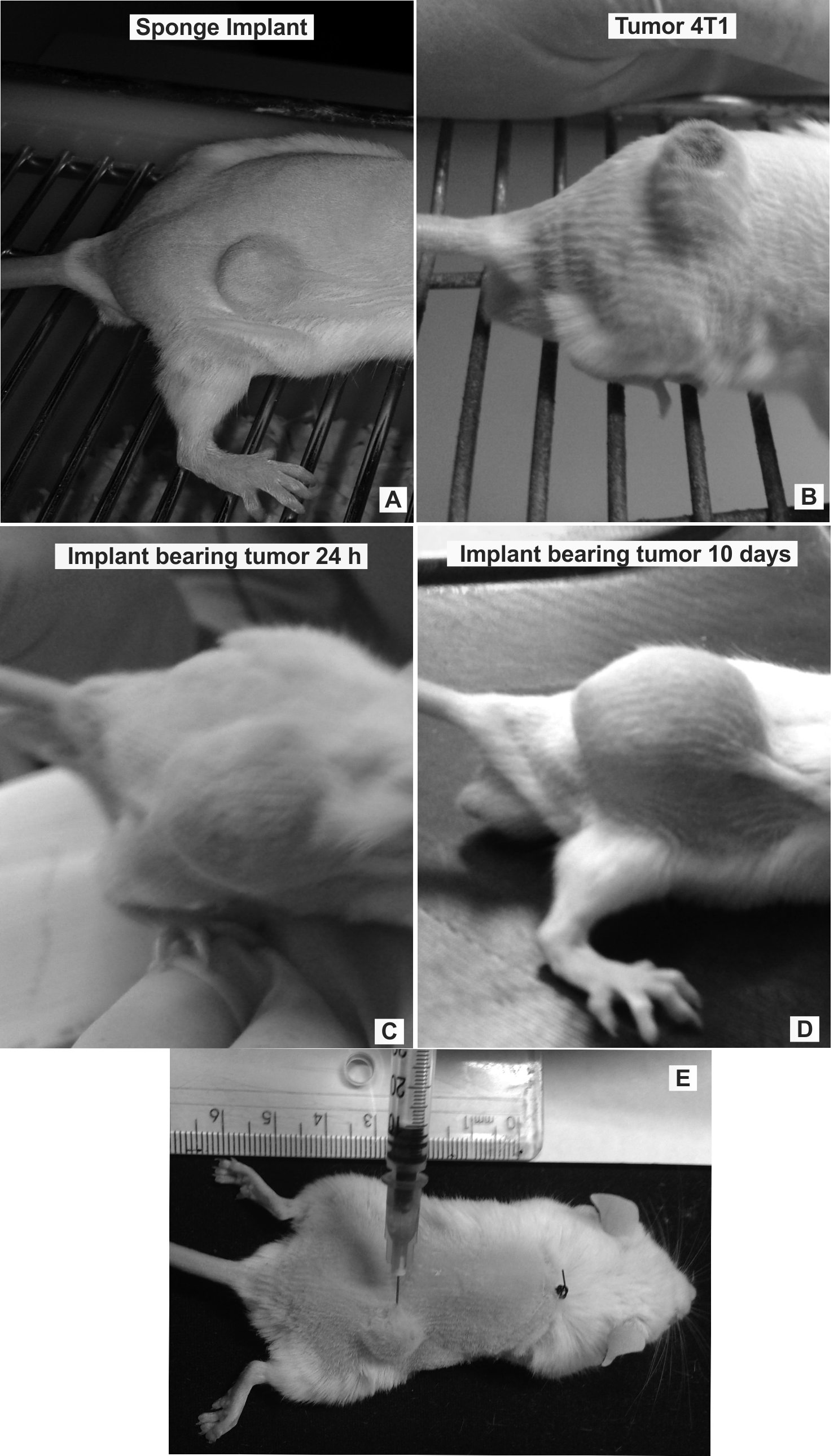

Supplement: S2 Fig — In A, sponge implant, in B, tumor alone; in C, tumor cells were inoculated in a 24 h-implant; in D, tumor cell were inoculated in a 10-day old implant. (TIF) [file pone.0130809.s003.tif]

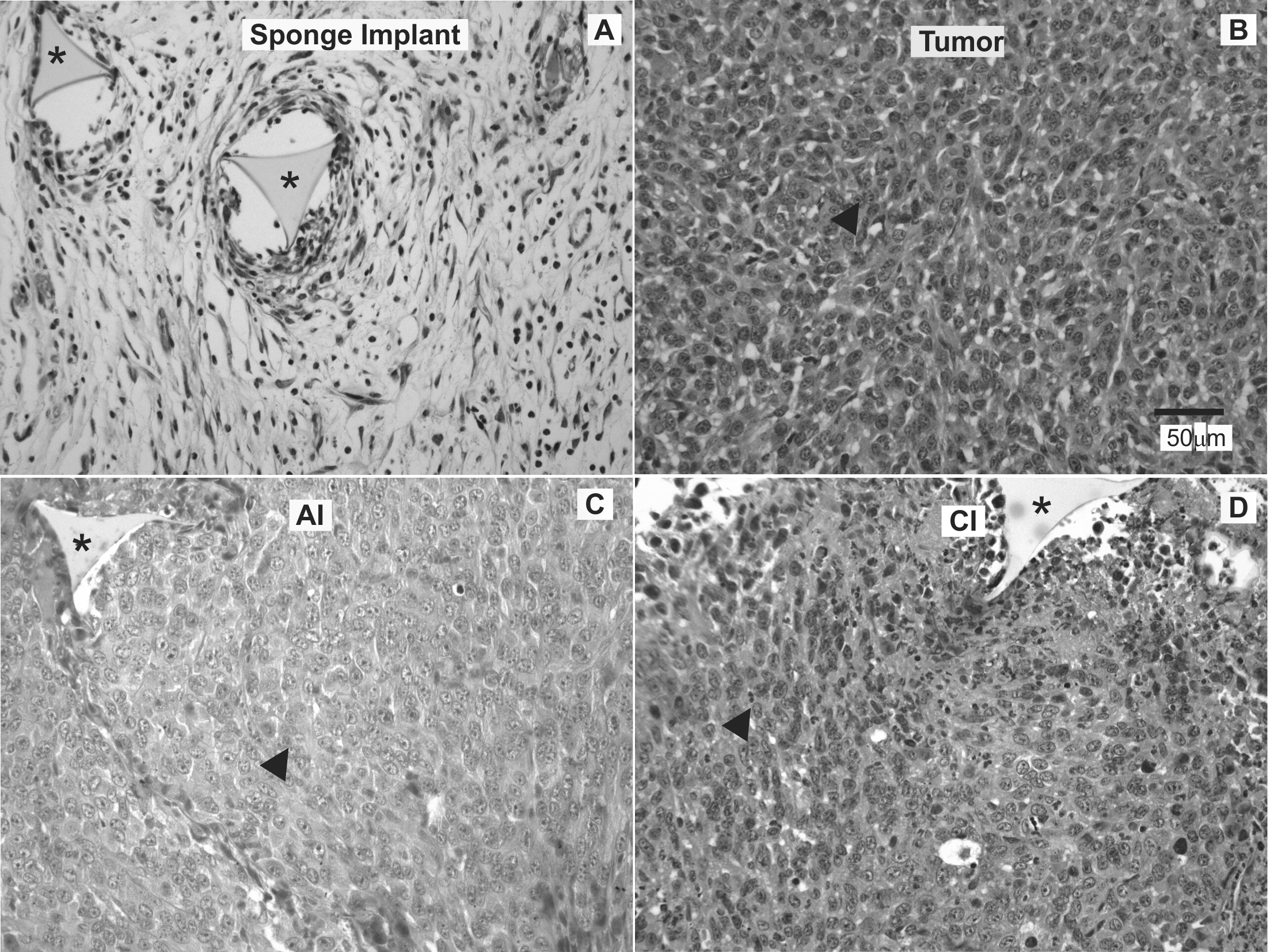

Supplement: S3 Fig — In tumor sections with or without the support of the synthetic matrix, neoplastic cells occupies the implant compartment along with blood vessels, inflammatory cells and stroma (B-D). Scale bar, 50 μm; * matrix; arrows, tumor cells. (TIF) [file pone.0130809.s004.tif]
